# Postoperative Functional Management Contributes to Anal Functional Recovery in Patients With Low Rectal Cancer After Robotic Total Intersphincteric Resection

**DOI:** 10.3389/fonc.2020.01373

**Published:** 2020-08-21

**Authors:** Wang Xiaosong, Liu Hongchang, Deng Min, Xie Lijuan, Li Chuan, Yu Peiwu, Tang Bo

**Affiliations:** ^1^Department of General Surgery, Southwest Hospital Affiliated to Army Medical University, Chongqing, China; ^2^Department of Rehabilitation, Southwest Hospital Affiliated to Army Medical University, Chongqing, China

**Keywords:** intersphincteric resection (ISR), low rectal cancer, anal function, biofeedback, robotic

## Abstract

**Purpose:** To evaluate the effectiveness of the comprehensive post-operative management including low-frequency endo-anal electrical stimulation and daily suppository usage on post-operative anal functional recovery for low rectal cancer patients who underwent robotic total intersphincteric resection (ISR).

**Methods:** A retrospective analysis was performed on 42 low rectal cancer patients who underwent robotic total ISR, of which 23 patients received comprehensive post-operative management, including biofeedback low-frequency endo-anal electrical stimulation and daily suppository usage (management group). Wexner score and anorectal manometric values, including resting pressure (RP), maximum squeeze pressure (MSP), initial perceived volume (IPV), and maximum tolerated volume (MTV), were assessed and compared.

**Results:** A total of 42 low rectal cancer patients were included in our study. The RP at 6 months after ISR (40.95 ± 6.95 mmHg vs. 33.29 ± 5.40 mmHg, *p* = 0.002) and MSP at 3 and 6 months after ISR (72.05 ± 10.16 mmHg vs. 69.05 ± 8.67 mmHg, *p* = 0.031; 91.57 ± 15.47 mmHg vs. 84.05 ± 12.94 mmHg, *p* = 0.039, respectively) were significantly higher in the management group. The median IPV at 1 and 3 months after ISR (17.81 ± 3.61 ml vs. 15.43 ± 5.08 ml, *p* = 0.038; 20.19 ± 4.35 ml vs. 17.67 ± 5.16 ml, *p* = 0.044, respectively) and MTV at 3 months after ISR (83.71 ± 5.44 ml vs. 76.10 ± 8.42 ml, *p* = 0.012) were significantly higher in the management group. Wexner scores at 1 and 3 months after closure of stoma (COS) in the management group were significantly lower (11.3 ± 2.9 vs. 13.4 ± 3.0, *p* = 0.041; 8.9 ± 2.0 vs. 10.6 ± 2.4, *p* = 0.036, respectively).

**Conclusions:** Comprehensive post-operative management could accelerate the recovery of sphincteric function and anal sensitivity after robotic total ISR and could also contribute to treatment of fecal incontinence followed by COS.

## Introduction

Abdominoperineal resection (APR) is a standard procedure for the curative resection of low rectal cancer. However, the unavoidable permanent stoma following APR has a negative effect on patients' self-esteem and quality of daily life (1). Innovative treatment for low rectal cancer has tended toward preservation of the anus. With the minimal invasive procedure including laparoscopy and robotic system widely used in the resection of low rectal cancer, the intersphincteric resection (ISR) with coloanal anastomosis becomes one of the most popular approaches for anus-preserving rectal cancer resection, despite the need for a temporary diverting stoma. However, the camera tremor and non-articulated forceps of laparoscopy make laparoscopic ISR technically demanding and restricted to some centers. The robotic system has been adopted in overcoming these disadvantages and made it easier to perform ISR. According to the resected grade of the internal sphincter, total ISR is defined as ISR at the intersphincteric groove, subtotal ISR is defined as ISR between the dentate line and intersphincteric groove, and partial ISR is defined as ISR at the dentate line (2). Evidence suggests that the oncological outcomes are similar to those following APR (3, 4). Meanwhile, the anal functional outcome has been indicated to be acceptable, especially for partial and subtotal ISR. Total ISR is performed for extremely low rectal cancer, and the incidence rate of anal dysfunction, resulting in a conversion to colostomy and reduced quality of daily life, is relatively higher with total ISR than with subtotal or partial ISR (5). Previous studies indicated that the total ISR group showed higher Wexner scores and bowel frequency compared with the partial and subtotal ISR groups (6, 7). Anal dysfunction after total ISR is mainly attributed to resection of the internal sphincter muscle and sphincteric or pudendal nerve damage during the operation. Hence, improvements in external sphincter muscle and levator ani muscle function represent a core aim for these patients. Electrical stimulation, especially low-frequency electrical biofeedback treatment, has been proven useful and is regarded as the first-line treatment for patients with fecal incontinence. Swash (8) conducted a prospective study evaluating the effectiveness of electrical stimulation on treating fecal incontinence and found it useful. Meanwhile, biofeedback was proven efficient in increasing the power and endurance of external anal sphincter contraction, and biofeedback enhanced the outcome of treatment compared with electrical stimulation or exercise alone (9). Norton and Cody (10) reported that biofeedback was effective for treating fecal incontinence and significantly improved sphincter muscle tone and squeeze pressure. In addition, anal suppositories have been used to form a protective film upon the rectum mucosa, improving anal irritation, and have been used for the treatment of fecal incontinence (11). Given that the fecal incontinence after robotic ISR is mainly attributed to resection of the internal sphincter muscle, we proposed comprehensive post-operative management, including biofeedback low-frequency endo-anal electrical stimulation and daily suppository usage, for patients who received robotic total ISR to evaluate the anal functional recovery efficacy.

## Materials and Methods

### Patients

A retrospective analysis of all the 42 patients undergoing total ISR from January 2016 to December 2017 at our institution was carried out in this study. All patients received robotic total ISR with a temporary diverting ileostomy. Our total ISR indications included a distance between the lower edge of the tumor and Hilton line ≥ 1 cm, moderately well-differentiated adenocarcinoma confirmed histologically by preoperative biopsy, suspected internal sphincter invasion (estimated stage cT_1−2_N_0−2_M_0_) and non-external sphincter infiltration according to preoperative enhanced rectal magnetic resonance imaging (MRI) and endoscopic ultrasonography evaluation, age <70 years, and normal anal sphincteric function. All patients were recommended to receive biofeedback low-frequency endo-anal electrical stimulation after surgery. Of these 42 patients, 23 patients received biofeedback management (the management group) and the other 19 patients did not (the control group).

### Surgical Procedure

Total ISR was performed by robotic surgical system according to a previously reported method (12). First, dissection was performed by the abdominal approach to the level of the pelvic floor. Then, the levator ani muscle hiatus was entered, and a division was created between the loose internal and external sphincter spaces to the level of the dentate line. Then, pelvic dissection was completed. The procedure was continued by transanal dissection. A circumferential incision of the mucosa and the internal anal sphincter was performed immediately at the Hilton line. With careful circumferential dissection and protection of the external anal sphincter and levator ani muscle, confluence at the level of the abdominal dissection and total ISR was completed. After transection of the specimen, reconstruction of bowel continuity was performed using an end-to-end procedure *via* a handsewn coloanal anastomosis with absorbable interrupted sutures.

### Postoperative Management

Biofeedback low-frequency endo-anal electrical stimulation was performed. The patients received endo-anal electrical stimulation in the rehabilitation department at our hospital using a preprogrammed biofeedback neuro-functional reconstruction system (AM1000A, Shenzhen, China) with an Anuform probe for 20 min twice daily for 3 months. Here, we had a preset biofeedback program designed with sequential frequencies of 20 Hz and with a current of 6 s on, once the voluntary contraction is detected, and 15 s off. The current intensity varied with the patients' sensitivity. Before the low-frequency electrical stimulation biofeedback therapy, every patient received instructions and training on the biofeedback treatment, the instruments, and how to cooperate with the therapists. The biofeedback stimulator was operated by two experienced biofeedback therapists (Dr. Deng Min and Dr. Xie Lijuan) in our hospital. Compound carraghenates suppositories were used daily.

### Assessment of Anal Function

Anal function and fecal incontinence were assessed using the Wexner score (13). Anorectal manometry was performed before and at 1, 3, 6, and 12 months after ISR by high-resolution manometry, and resting pressure (RP), maximum squeeze pressure (MSP), initial perceived volume (IPV), and maximum tolerated volume (MTV) values were assessed to evaluate sphincteric and fecal functions. Wexner scores were used to evaluate the fecal incontinence before ISR and every 3 months after closure of stoma (COS).

### Statistical Analysis

Categorical data are presented as the number of cases evaluated, and quantitative data are reported as the mean ± standard deviation (SD). The manometry values and Wexner scores after ISR were statistically analyzed by Student's *t-*test and Fisher's exact test. Patients in this study were divided into two groups according to post-operative comprehensive management following ISR: a management group and a control group. All statistical analyses were performed by using SPSS 22.0 (SPSS Inc., Chicago, IL, USA). *P* < 0.05 were regarded as statistically significant. The combination of anal contraction training and suppository usage was used to train the anal control function. Suppositories were used twice daily, and conscious sphincteric contraction was performed at any time.

## Results

A total of 42 low rectal cancer patients who received robotic total ISR were included in this study. Of these patients, 19 were women, and 23 were men. Of the 42 patients, 23 received comprehensive post-operative management, and the remaining 19 patients did not. The characteristics and perioperative clinical details are shown in Table 1. There were no significant differences in these characteristics between these two groups. All patients received stoma closure 3–6 months after ISR, and none of these patients received the conversion to permanent colostomy for the intolerability of fecal incontinence.

**Table 1:** Demographics and treatment details.

	**Control**		**Management**		***t-*value**	***P-*value**
	**Group**		**Group**			
	**Mean**	**SD**	**Mean**	**SD**		
Age	53.43	9.80	54.10	10.29	−0.215	0.672
Margin distance (cm)	2.02	0.44	2.01	0.28	0.084	0.070
Tumor size (cm)	1.69	0.49	2.31	0.60	2.618	0.114
Lymph node dissection	11.14	5.35	13.33	3.15	−1.616	0.066
Blood loss (ml)	60.71	23.68	65.95	19.60	0.305	0.584
BMI (kg/m^2^)	24.77	3.72	24.71	2.15	0.236	0.792

*Margin distance means the distance between tumor and intersphincteric groove*.

**Figure 1:**
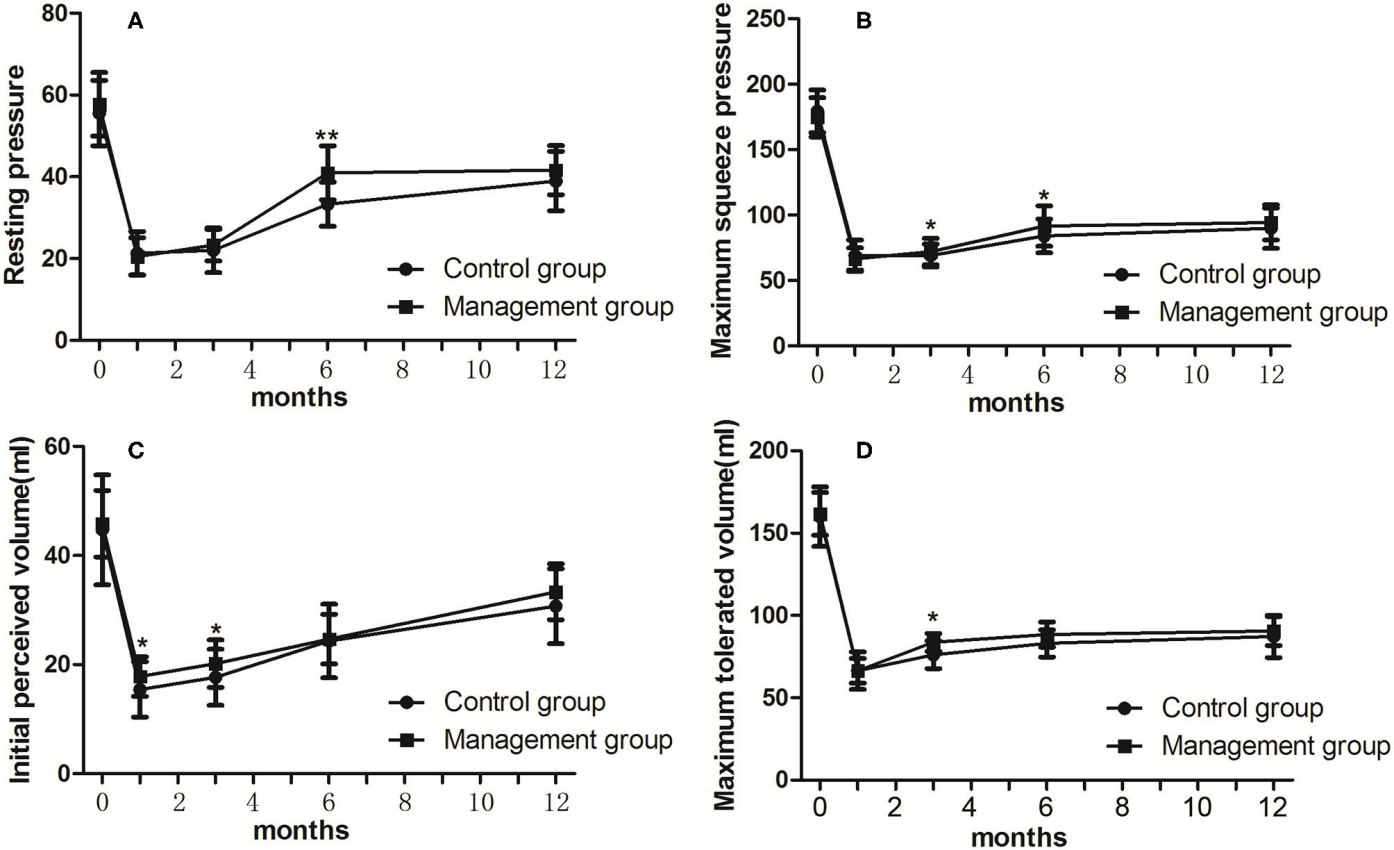
Differences of pre- and post-operative values (**A** for RP; **B** for MSP; **C** for IPV; **D** for MTV) of manometry between groups. RP, resting pressure; MSP, maximum squeeze pressure; IPV, initial perceived volume; MTV, maximum tolerated volume. *p < 0.05, **p < 0.01.

To evaluate post-operative anal function objectively, we measured some functional values, including RP, MSP, IPV, and MTV, before and 1, 3, 6, and 12 months after the operation. We regarded the preoperative manometric values as the baseline values and evaluated the difference in these values after ISR. The preoperative median RP, MSP, IPV, and MTV values were 56.45 mmHg, 177.02 mmHg, 45.26 ml, and 160.88 ml, respectively (Table 2). These values were significantly decreased after total ISR and slowly recovered as time progressed. We also assessed the clinical efficacy of our comprehensive management by evaluating the difference in values between the control group and management group, which indicated that the median RP at 6 months after ISR (40.95 ± 6.95 mmHg vs. 33.29 ± 5.40 mmHg, *p* = 0.002) and the median MSP at 3 and 6 months after ISR (72.05 ± 10.16 mmHg vs. 69.05 ± 8.67 mmHg, *p* = 0.031; 91.57 ± 15.47 mmHg vs. 84.05 ± 12.94 mmHg, *p* = 0.039, respectively) were significantly higher in the management group. These results indicate that comprehensive management accelerated sphincteric functional recovery after total ISR (Table 3; Figures 1A,B). We also found that the median IPV at 1 and 3 months after ISR (17.81 ± 3.61 ml vs. 15.43 ± 5.08 ml, *p* = 0.038; 20.19 ± 4.35 ml vs. 17.67 ± 5.16 ml, *p* = 0.044, respectively) and the median MTV at 3 months after ISR (83.71 ± 5.44 ml vs. 76.10 ± 8.42 ml, *p* = 0.012) were significantly higher in the management group (Table 3; Figures 1C,D).

**Table 2:** Values of manometry before and after total ISR.

	**Preoperative**	**1 month**	**3 months**	**6 months**	**12 months**
RP (mmHg)	56.45 ± 7.84	20.93 ± 4.91^**^	22.67 ± 4.68^**^	37.12 ± 9.10^**^	40.29 ± 6.72^**^
MSP (mmHg)	177.02 ± 15.60	67.69 ± 10.33^**^	70.55 ± 10.54^**^	87.81 ± 14.14^**^	92.12 ± 14.28^**^
IPV (ml)	45.26 ± 8.25	16.62 ± 4.52^**^	18.93 ± 4.89^**^	24.50 ± 5.68^**^	32.02 ± 6.43^**^
MTV (ml)	160.88 ± 15.57	66.45 ± 9.55^**^	79.90 ± 8.88^**^	85.71 ± 9.46^**^	88.90 ± 10.18^**^

*ISR, intersphincteric resection; RP, resting pressure; MSP, maximum squeeze pressure; IPV, initial perceived volume; MTV, maximum tolerated volume*.

** *p < 0.01 (compared with preoperative)*.

**Table 3:** Pre- and post-operative values of manometry based on different groups.

	**Control Group**		**Management Group**		***t-*value**	***P-*value**
	**Mean**	**SD**	**Mean**	**SD**		
RP (pre-) (mmHg)	55.57	8.00	57.33	7.76	−0.724	0.473
RP (1) (mmHg)	21.33	5.31	20.52	4.56	0.530	0.599
RP (3) (mmHg)	22.05	5.45	23.29	3.86	−0.522	0.604
RP (6) (mmHg)	33.29	5.40	40.95	6.59	−4.124	0.002^**^
RP (12) (mmHg)	38.95	7.26	41.62	6.00	−1.297	0.202
MSP (pre-) (mmHg)	179.33	16.25	174.71	14.95	0.959	0.344
MSP (1) (mmHg)	68.90	11.97	66.48	8.51	0.758	0.453
MSP (3) (mmHg)	69.05	8.67	72.05	10.16	−2.921	0.031^*^
MSP (6) (mmHg)	84.05	12.94	91.57	15.47	−2.563	0.039^*^
MSP (12) (mmHg)	89.90	15.40	94.33	13.45	−1.096	0.924
IPV (pre-) (ml)	44.71	10.09	45.81	6.09	−0.426	0.672
IPV (1) (ml)	15.43	5.08	17.81	3.61	−1.751	0.038^*^
IPV (3) (ml)	17.67	5.16	20.19	4.35	−1.713	0.044^*^
IPV (6) (ml)	24.33	6.74	24.67	4.54	−0.188	0.852
IPV (12) (ml)	30.71	6.88	33.33	5.12	−1.332	0.190
MTV (pre-) (ml)	160.05	18.08	161.71	12.99	−0.343	0.733
MTV (1) (ml)	66.47	11.4	66.42	7.55	0.016	0.987
MTV (3) (ml)	76.10	8.42	83.71	5.44	−3.586	0.012^*^
MTV (6) (ml)	83.04	8.27	88.38	7.63	−1.631	0.111
MTV (12) (ml)	87.23	12.89	90.57	8.79	−0.610	0.635

*RP (pre-), preoperative RP; ISR, intersphincteric resection; RP, resting pressure; MSP, maximum squeeze pressure; IPV, initial perceived volume; MTV, maximum tolerated volume; RP (x), RP of x months after ISR*.

* *p < 0.05*,

** *p < 0.01. Bold values means statistically significant*.

**Figure 2:**
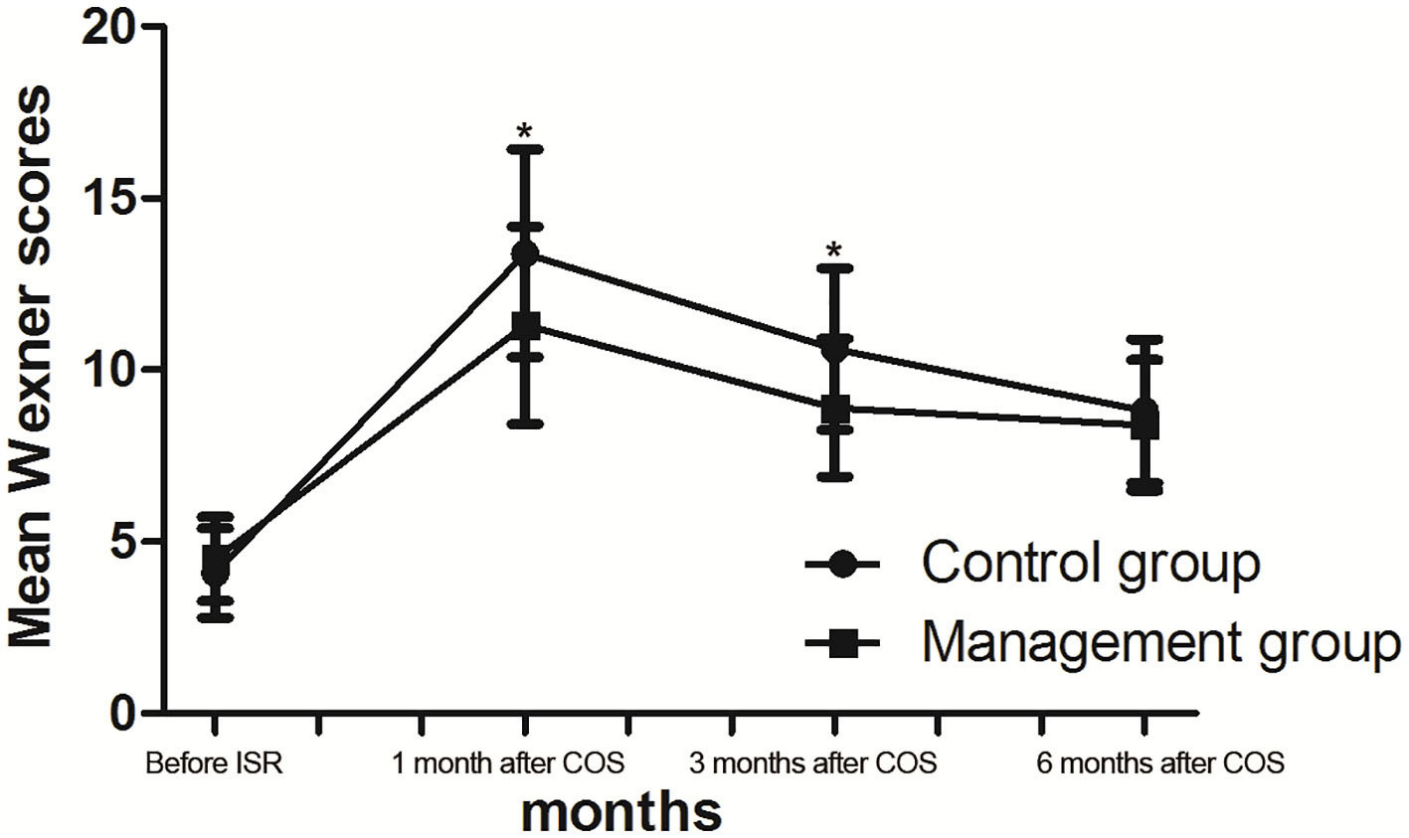
Wexner scores (mean) before intersphincteric resection (ISR) and after closure of stoma (COS). *p < 0.05.

The Wexner scores were significantly increased after stoma closure in both groups after COS (Table 4). The mean pre-ISR and post-COS (1, 3, and 6 months) Wexner scores were 4.1, 13.4, 10.6, and 8.8 in the control group and 4.5, 11.3, 8.9, and 8.4 in the management group, respectively. The Wexner scores in the management group were significantly lower than those in the control group at 1 and 3 months after COS, while there is no significant difference 6 months after COS (Figure 2).

**Table 4:** Wexner scores (mean) before intersphincteric resection (ISR) and after closure of stoma (COS).

	**Before ISR**	**1 month after COS**	**3 months after COS**	**6 months after COS**
Control group	4.1 ± 1.3	13.4 ± 3.0^**^	10.6 ± 2.4^**^	8.8 ± 2.1^**^
Management group	4.5 ± 1.2	11.3 ± 2.9^**^	8.9 ± 2.0^**^	8.4 ± 1.9^**^

** *p < 0.01 (compared with preoperative)*.

## Discussion

Sphincteric dysfunction is a primary cause for conversion to permanent colostomy for patients with rectal cancer after total ISR. To increase anal function and reduce the incidence of fecal incontinence after robotic total ISR, we proposed a comprehensive management procedure, including low-frequency anal electrical biofeedback treatment and daily endo-anal suppository usage. It was reported that low-frequency anal electrical stimulation could improve motor activity and increase sensory feedback, hence significantly increasing fatigue resistance and improving squeeze pressure (14). Biofeedback is an effective and non-invasive treatment for fecal incontinence and has been regarded as a first-line therapy for fecal incontinence with satisfactory clinical outcomes (15). Electromyographic biofeedback could render the sphincter muscle contraction visible by plotting the contractions on a graph, from which patients and therapists can identify when the contraction begins, how long it lasts, and when it is over, resulting in easier acceptance of the treatment and better cooperation with therapists. The suppositories are widely used for anorectal disease, including fecal incontinence (11). Given that the electrical biofeedback may trigger the rectal mucosa edema or damage, and the compound carraghenates suppositories were reported to be useful for reducing rectal mucous injury from acetic acid and accelerating wound healing and that they can relieve pain and improve mucosal edema (16, 17), and the compound carraghenates suppositories could form a protective film upon the rectum mucosa to avoid the stimulation from electrical biofeedback treatment, we combined electrical biofeedback treatment with daily compound carraghenates suppository usage for patients who underwent total ISR to increase sphincteric function and anal sensitivity. RP and MSP objectively reflect sphincteric function (18). In this study, RP and MSP were significantly reduced after total ISR, indicating that the injury of anal function after resection of the internal sphincter muscle could be reflected by the RP and MSP values. We also found that RP and MSP were significantly higher in the management group 3–6 months after total ISR, even though there was no significant difference at 12 months after surgery. This result indicated that our comprehensive management could accelerate sphincteric functional recovery after total ISR. It was reported that the internal sphincter muscle contributes ~ 55% of the total anal pressure, while the external sphincter muscle contributes ~ 30% (19). In this study, the robotic total ISR completely removed the internal sphincter muscle, which means the removal of approximately half of anal function. In the comprehensive management group, the electromyographic biofeedback treatment enhanced the compensatory function of the external sphincter muscle, and suppository usage improved the side effects of electromyographic biofeedback treatment and enhanced anal contraction training efficacy, resulting in the acceleration of external sphincteric functional recovery.

In addition, the IPV and MTV were used to evaluate anal sensitivity (8). In this study, the median IPV and MTV were significantly decreased and then slowly recovered after total ISR. In the management group, the IPV and MTV were higher than those in the control group 1–3 months after surgery. Compared with RP and MSP, the recovery of the IPV and MTV occurred earlier in the management group than in the control group, indicating that comprehensive management likely contributes more to the recovery of anal sensitivity. We hypothesized that these effects might be due to electromyographic biofeedback stimulation of the hemorrhoidal plexus and suppository usage, which could provide a foreign body sensation and unconsciously induce sphincteric contraction. The decrease in the MTV suggested that fecal incontinence after total ISR was urge incontinence, which is a common type of functional or neuropathic fecal incontinence. From this study, we found that sphincteric function and anal sensitivity could partially recover naturally after total ISR. The anal function could recover to a relatively satisfying level ~ 12 months after ISR. The results showed that the post-operative management including electromyographic biofeedback and suppository usage could accelerate the anal function recovery by ~ 3–9 months.

The Wexner score is the most widely used method for evaluating anal function after ISR (3, 7, 20). Quality of life could be partially reflected in the Wexner scores. The improvement of fecal continence reflected in the Wexner scores may be related to anal sensory awareness and self-confidence. In this study, the Wexner scores were significantly increased after COS in both groups (Table 4). In addition, the Wexner scores were significantly lower both at 1 and 3 months after COS in the management group than in the control group after COS, suggesting that comprehensive management is helpful for rectal sensitivity and anal function recovery after total ISR. We also found that even though manometric values were obviously different, the Wexner scores tended to adience as time prolongs, and 6 months after COS the Wexner scores was not significantly different, indicating that anal manometry could partially reflect the anal function. The Wexner scores consisted of some subjective feeling parameters, which indicated that the subjective feeling of the patients should be considered an important factor in anal function evaluation. In addition, patients would adjust their daily diet based on their defecation condition.

In addition to electromyographic biofeedback and suppository usage, there are other factors, including exercise and diet, that could affect anal function after total ISR. Based on communication with these patients, we realized that most patients would rather bear increased defecation frequency rather than the permanent stoma.

In this study, the biofeedback process was time-consuming and demanded visits to the Department of Rehabilitation at our hospital throughout the whole period of treatment. In fact, this comprehensive management was recommended to all of these 42 patients, while the individuals in the control group in our study refused this management program mostly due to the inconvenience of constant hospital attendance and were included into the control group. Some studies have attempted to construct a biofeedback device specifically developed for home treatment, and these devices have been proven useful (19, 21–23). We are also attempting to identify a safe and convenient household electrical stimulator to accomplish electromyographic biofeedback therapy at home.

In conclusion, comprehensive post-operative management could accelerate the recovery of sphincteric function and anal sensitivity after robotic total ISR and could contribute to the treatment of fecal incontinence after COS. Our study had some limitations. First, this was not a prospective randomized controlled study, and there may be some potential bias in patient inclusion. The effectiveness of this management plan for total ISR should be further clarified in future studies. Second, the relatively small size of the study sample might weaken the statistical power of this study. Third, in-depth studies should be performed to explore the most valuable management protocol to obtain the most benefits for patients.

## Data Availability Statement

The raw data supporting the conclusions of this article will be made available by the authors, without undue reservation.

## Ethics Statement

The studies involving human participants were reviewed and approved by the institutional review board of the Southwest Hospital Affiliated to Army Medical University. The patients/participants provided their written informed consent to participate in this study.

## Author Contributions

WX and LH wrote the main manuscript text. DM, XL, and LC prepared the tables and figures. YP and TB revised and approved the manuscript text. All authors reviewed the manuscript. All authors contributed to the article and approved the submitted version.

## Conflict of Interest

The authors declare that the research was conducted in the absence of any commercial or financial relationships that could be construed as a potential conflict of interest.
